# EvacuAI: An Analysis of Escape Routes in Indoor Environments with the Aid of Reinforcement Learning

**DOI:** 10.3390/s23218892

**Published:** 2023-11-01

**Authors:** Anna Carolina Rosa, Mariana Cabral Falqueiro, Rodrigo Bonacin, Fábio Lúcio Lopes de Mendonça, Geraldo Pereira Rocha Filho, Vinícius Pereira Gonçalves

**Affiliations:** 1 Electrical Engineering Department, University of Brasilia, Brasilia 70910-900, DF, Brazilfabio.Mendonça@redes.unb.br (F.L.L.d.M.); vpgvinicius@unb.br (V.P.G.); 2 UNIFACCAMP and CTI Renato Archer, Campinas 13069-901, SP, Brazil; rodrigo.bonacin@cti.gov.br; 3 Department of Exact and Technological Sciences, State University of Southwest Bahia (UESB), Vitória da Conquista 45083-900, BA, Brazil; geraldo.rocha@uesb.edu.br

**Keywords:** machine learning, deep reinforcement learning, transfer learning, fire, evacuation

## Abstract

There is only a very short reaction time for people to find the best way out of a building in a fire outbreak. Software applications can be used to assist the rapid evacuation of people from the building; however, this is an arduous task, which requires an understanding of advanced technologies. Since well-known pathway algorithms (such as, Dijkstra, Bellman–Ford, and A*) can lead to serious performance problems, when it comes to multi-objective problems, we decided to make use of deep reinforcement learning techniques. A wide range of strategies including a random initialization of replay buffer and transfer learning were assessed in three projects involving schools of different sizes. The results showed the proposal was viable and that in most cases the performance of transfer learning was superior, enabling the learning agent to be trained in times shorter than 1 min, with 100% accuracy in the routes. In addition, the study raised challenges that had to be faced in the future.

## 1. Introduction

Serious fires in buildings are continuing to break out throughout the world, despite advances in fire safety standards, technology, and protective legislation. Fire usually spreads quickly in indoor environments, which means the reaction time to a fire threat from the moment an alarm is sounded is very short, ranging from seconds to a few minutes [1]. As soon as the best way out of the building has been determined, there will be a greater chance of escaping without injury. Unfortunately, finding the best way out in these cases is a complex task, owing to the rapid speed and intensity of fires and the tendency of people to panic.

According to the Occupational Safety and Health Administration (OSHA) in the U.S.A., an emergency exit route can be defined as a continuous, unobstructed path from any point within a workplace to a safe location. These routes should be taken by people during an emergency (e.g., fire) to get away from a dangerous area to a safe place [2]. Emergency exit routes are extremely important to ensure the safety of buildings, since they provide a quick and safe means of escaping from a dangerous situation. Moreover, these routes are needed for firefighters, first responders, and the police during the rescue operation.

Data from Brazil’s Unified Health Unit System (Note: Brazil’s Health Unit System is one of the largest public health systems in the world. It is designed to provide universal and free access to simple and complex healthcare to the whole of the country) show that there were more than 1050 deaths from fire or exposure to smoke in Brazil in 2011, which means the country had the third highest number of deaths from fire in the world [3]. In Brazil, the local construction standards (NBR 9077) regulate and list all the necessary specifications to ensure the safety of a building, including emergency exit routes [4]. In 2020, the United States Federal Agency Fire Administration reported an estimated number of 372,000 residential fires and 2615 deaths [5].

In general, all buildings must have a fire escape/evacuation plan, in which it is imperative that there are at least two recognizable emergency exit routes. In addition, smoke sensors must be connected to fire alarm warning systems to assist the firefighters. As a result of advances in technology, it is now possible to create applications to assist in the evacuation of people from a building in the event of a fire. These applications may, for instance, be used to analyze the building floor plans, together with the smoke sensors connected to the fire alarm systems [6].

The literature shows several research studies on how the emergency exit routes can be found quickly. Usually, these studies are based on an analysis of the floor plans of the buildings, which are represented in either a graph or matrix (e.g., in [7,8,9]). However, the automated analysis and labeling include challenging computational tasks, since they require precise techniques to replace or support complex manual processes.

If there are already computational representations of a building floor plan, path-finding algorithms to trace emergency exit routes can be used, relying on well-known algorithms such as Dijkstra, Bellman–Ford, and A* [10]. However, these algorithms can lead to serious problems in performance, as the number of nodes in the graph (which represents the building floor plan) grows.

These well-known algorithms are usually focused on a single objective, such as finding the shortest path between two nodes. However, for multi-objective problems, such as those being addressed in this study, it is essential to find not only the shortest path but also one that avoids a blocked node (hit by fire). In this regard, reinforcement learning (RL) algorithms stand out as they allow the incorporation of more objectives for analysis in the future, such as fire spread, bottleneck identification, and analysis of the number of people at each node, to achieve a better path distribution [11]. Thus, it is necessary to investigate the use of advanced computing techniques, such as RL [12].

RL [12] is a sub-field of unsupervised machine learning, which is based on the principle of granting rewards for desired behavior and punishments for what is undesirable. In this technique, an RL agent interprets its environment and carries out actions, and learns by means of a trial and error strategy, and by always seeking to maximize its final reward. As well as often being used in game scenarios, RL can also be used for best-path search in graphs and matrices that represent real-world situations.

Thus, the objective of this work is to investigate alternative ways of ensuring the rapid evacuation of people in fire outbreaks in indoor environments on the basis of RL. RL techniques are still under-explored in real-time and real-world applications that require fast training as well a high degree of precision and accuracy in their results. By representing the floor plan of an indoor building in a graph, the best exit routes can be found in a few seconds or minutes.

This work makes a research contribution by offering a complete solution and assessment of the problem outlined above. This solution involves employing a tool and model for assisting in the manual construction of graphs that represent the floor plans of indoor environments, where the nodes will be the rooms and the edges will be the connections between the rooms (e.g., doors, doorways and windows that can be used as part of a route). Following this, an RL algorithm uses this representation to discover, in real-time, the best emergency exit routes in the event of fire.

We decided to use deep reinforcement learning (DRL) since it is able to solve more complex problems than RL. In DRL, a neural network is used to estimate the states instead of mapping all the solutions, with the aim of achieving a better result and adopting a faster decision-making process.

Three methods were used to assess the performance of the DRL model. These methods rely on experience replay and the target network, the first initializes the replay buffer with random values, whereas the second does not initialize them, and the third adopts a transfer learning strategy. Section 2 outlines these methods.

The remainder of this article is structured as follows: Section 2 defines concepts and describes the background. Section 3 presents related work on graph-based representations of floor plans and on the use of RL (and DRL) for searching for the shortest path within an environment. Section 4 describes the proposed solution and the application employed to deal with the question of the emergency exit routes. Section 5 discusses the experiments carried out with the proposed application, and Section 6 wraps up the the study in conclusion and makes suggestions for further research.

## 2. Background

Most studies on machine learning are focused on supervised learning. To master this technique it is necessary to label and map the training input and output data, i.e., the system learns from pre-defined correct maps in order to predict correct answers through new inputs. This technique usually depends on human intervention for the creation and labeling of training maps. However, this is not a feasible solution when there is a need to learn by interacting with a dynamic environment, where several different situations have not been previously mapped. Hence, a technique that does not need pre-defined mapping (e.g., RL) is required for them [13].

However, with the unsupervised learning technique, human intervention is not necessary, since it adopts an approach which enables it to explore unknown data. RL cannot be considered either supervised or unsupervised, and can be considered as the third paradigm of ML, based on the trial and error method, which seeks to learn how to maximize the reward for a specific task. Since the learning agent is not informed about the best action it should take, it has to interact with the environment to discover the action and, thus, maximize its reward. By interacting with the environment and gathering experiences, a learning agent is able to (a) detect patterns, (b) understand and process the data, and (c) learn for itself the best actions to take [13].

The policies of exploration and exploitation are key concepts of RL [14]. An RL agent uses them to assess its past experiences and decide which actions to take. If it always decides to take actions that have already been chosen, it will not explore all the possible actions within the environment. Hence, an RL agent must also explore the environment by choosing actions that have not previously been selected, to compare with the results that have already been obtained. Exploitation is a policy used by the RL agent to take actions based on its past experiences, and exploration is a policy used by the RL agent to randomly select actions, with a view to obtaining knowledge of the entire environment.

In this study, we have selected the six main elements of an RL method: the agent, the environment, the state, policy, reward, and value function [15].

The RL agent is the entity responsible for interacting and making decisions in the environment. Each time an agent carries out an action, it is in a “state” (*s*) that provides a reward (*r*). After each action, the agent will find itself in a new state ($s^{\prime }$) [15].

The environment is the space where the RL agent carries out its activities. In this work, the environment is represented by a graph and its adjacent matrix. Graphs are a branch of mathematics that are designed to study the relationship between the objects of a given set. A simple graph can be represented as:

(1) G=(V,A),

where *V* is considered to be a “non-empty” set of objects known as nodes and *A* a subset of unordered pairs of *V*, known as edges, so:

(2) A⊆P(V)andP(V)={{x,y}:x,y∈V}.

In a non-reflexive graph, each edge is formed of a pair of distinct nodes, thus *x ≠ y* [16].

The policy (π) is the strategy used by an agent at a given moment to decide the next action. The stochastic probability pattern is frequently used to this end. The policy can be regarded as the core of an RL system, as it is responsible for determining the agent’s behavior [15].

The reward (*r*) is used to indicate how good the next action that can be taken is. Nonetheless, it is not able to define how good a (long-term) path from the start to a specific state will be. A value function must be used to this end.

The Bellman equation is one of the core elements of RL, since it uses value functions to define an equation formulated for the relationship between the value of a state and the values of the possible subsequent states. With the aim of simplifying the decision-making process, this equation calculates the average of all the possibilities, and uses the weight values in accordance with the probability of an occurrence.

Bellman’s equation states that the value of the initial state is equal to the discounted value of the expected next state plus the expected reward along the path, as defined in Equation (3) [17], where the gamma (γ), often called the discount factor, controls the importance of long-term versus immediate rewards.

(3) $$v_{\pi }\left(s\right)≐\sum_{a}\pi \left(a|s\right)\sum_{s^{\prime },r}p\left(s^{\prime },r|s,a\right)\left[r+\gamma v_{\pi }\left(s^{\prime }\right)\right],\mathrm{for}\;\mathrm{all}s\in S.$$

The Q-learning algorithm seeks to provide the best action to be taken given its current state. This algorithm is often used in RL implementations. Through this algorithm, Q*(s,a) will be the expected value to perform an action *a*, being in a state *s* following the optimal policy. The algorithm uses the technique of unsupervised temporal difference (TD), by which the RL agent learns to predict the expected value of a variable that occurs at the end of a sequence of states [18]. The RL agent keeps an updated table Q[S,A] of states and actions, known as the Q-table.

The Q-function is used to update the Q-table. As defined in Equation (4), this function uses the concepts of the Bellman equation.

(4) $$Q\left(S_{t},A_{t}\right)\leftarrow Q\left(S_{t},A_{t}\right)+\alpha \left[R_{t+1}+\gamma maxQ\left(S_{t+1},a\right)-Q\left(S_{t},A_{t}\right)\right].$$

The alpha (α) is a real number between 0 and 1, which represents the agent’s learning rate. The gamma γ is a real number between 0 and 1, known as the discount factor, which defines the importance of the next reward.

DRL is a sub-field of RL that combines RL with deep learning (DL) techniques. DRL is also known as deep Q-learning, because it is usually based on the Q-learning algorithm. This is the method used in this work.

Neural networks benefit RL, especially when there is a vast environment as well as a large number of actions to be taken by the agent, such as in the case of building floor plans. A neural network is used to given an approximation of the value functions or policy functions, as it learns to map the pairs of state–action, as performed by the Q-learning algorithm. In this case, the Q-table is replaced by a neural network.

Experience replay is one of the most widely used techniques in DRL, as it seeks to increase the efficiency and stability of the training. This technique uses a memory or repetition buffer of a fixed size to store the most recent transitions collected by the policy, that is, the most recent experiences lived by the agents. This data can be used several times during the training [19].

A deep Q-learning algorithm uses a neural network instead of an exact value function as Q-learning does. This is an attempt to approximate a complex non-linear function, which can end up making the network unstable and impairing the next “state” decisions that have to be made [20].

The mean squared error (MSE) loss function (used in this work) requires two values: the Q-predicted value (*s*) and the Q-target value ($s^{\prime }$). Thus, the deep Q-learning algorithm makes estimations of these values using two neural networks to avoid instability in predicting the Q-target value [21].

To avoid this kind of instability, an auxiliary network is used to estimate the Q-target. This auxiliary network, called the target network, is a copy of the original neural network, i.e., it has the same architecture. However, its weights are still unchanged (i.e., frozen) most of the time. It is adjusted at larger intervals in accordance with the weights of the original network to avoid changes in the Q-target prediction at each iteration.

The target networks stabilize the training, as they maintain a separate copy of the original neural network for calculating the value of the Bellman equation $Q(s^{\prime },a^{\prime })$. Since the values of the target network are used as a back propagation and training system of the main neural network, this duplication reduces the correlation between *s* and $s^{\prime }$ [20].

## 3. Related Works

The automated definition of emergency exit routes in indoor environments has been studied for many years. Numerous studies have been concerned with how to achieve a quick evacuation of places in an emergency, including fire. Some studies use machine learning algorithms to search for secure paths [8] or even information from a building information model (BIM), as well as images of the building, to define the best exit route in real time [22].

Reference [8] shows the use of the A* and k-means algorithms. K-means is an unsupervised machine learning algorithm based on clustering. The paper shows how these algorithms can be used to search for the best path in a matrix, which represents the building floor plan. Although this work does not explore the concept of fire propagation when searching for the best exit route, the method is a valuable asset in the work [8]. Unlike our study, the representation used in this reference is not suitable for real time because there is uncertainty about how to carry it out. However, this problem is not mentioned in the article and it is not really known if the solution can be applied to real-time cases.

Reference [23] proposed the use of a unity game engine in the analysis of emergency exit routes by simulating situations in a shopping center. An artificial intelligence algorithm based on the A* algorithm was used; although, it does not focus on real-time exit routes. The learning environment was created with computer aided design (CAD) tools, so it was possible to represent a real environment, as well as to obtain results and analysis very close to reality, including the existing obstacles in the shopping center.

By means of RL it is possible to train a machine learning algorithm with little information and a system of rewards. For this reason, the technique, which is used in our study, has been explored by some studies in the literature, as shown below.

With the aid of RL, Reference [24] proposes a system that relies on real-time information on escape routes in crime scenes. The purpose of this is to assist those involved in pursuit of the criminals. It is based on the Markov decision process and Q-learning, and thus differs from our system that uses deep neural networks. However, this reference does not show how a graph can be drawn from a crime scenario.

In [9], the Q-learning algorithm is used to find the best emergency exit route in an indoor environment. However, this study work differs from ours in several respects, including the use of deep neural networks (in our work) and the representation of the environment, which is based on binary values (0 or 1) in their work, which indicates if there is a free or blocked area in the environment.

Reference [7] recommends measuring the distance between the nodes to calculate the reward returned to the agent, in addition to the use of the experience replay method for DRL. In our work, graphs (to improve the performance) and adjacent matrices are used to represent the environment, and the transfer learning (TL) method and DRL are displayed for real-time decision making.

RL requires the construction of an environment in which the agent interacts. In this work, a graph will be used to represent the floor plans, from which the learning environment will be built. However, extracting the graph from an image of the floor plan is a challenging task that requires the use of image processing techniques, deep learning, or even manual annotations.

Reference [25] represents an image of a floor plan in a graph and employs image-processing techniques. In [26], the bitmap (pixel-by-pixel) of the image is analyzed. One problem with these techniques is that they can be inaccurate depending on the floor plan, since they are dependent on the quality of the image and how the floor plans were created and designed.

An alternative system is the use of machine learning to extract valuable information from an image of a floor plan, such as the rooms, walls, doors, and windows.

The aim of the Graph2Plan [27] project is to produce floor plans from user information, and the CubiCasa5k [28] and CubiGraph5k [29] projects are able to separate the floor plan designs of each room into polygons, and then create the corresponding floor plan graph. A serious drawback of the various current projects is that they are focused and trained on the floor plans of houses, with few rooms, i.e., they are simpler than the floor plans used in our work.

In [30], a mask region-based convolutional neural network (R-CNN) was used to evaluate the image bitmaps for the segmentation of the floor plans, for the automated construction of 2D BIM. They assessed around four thousand images. Good results were obtained when they were applied to floor plans with easy to medium difficulties, although the results were worse for images that were thought to be hard to evaluate [31].

As shown above, most of the cited works that seek to represent floor plans in graphs are focused on the floor plans of houses, i.e., simple plans with few nodes and edges. Research studies on the floor plans of large buildings are still needed. Large buildings usually have more complex floor plans, which require large datasets and increase the computational costs incurred for the task.

Thus, the literature raises several challenges for the automated generation of graphs from images of floor plans. An alternative is to provide a manually assisted process. Although this is a laborious task, it is expected to produce a model with a greater degree of accuracy than the methods discussed above. Table 1 summarizes the key features of the cited related works and the solution set out in this article. Our work differs from others since it is the only study to use DRL, to suggest exit routes in real time, and to build representations of training environments.

## 4. Proposed Solutions

This section describes the solution put forward for both problems as follows. Section 4.1 presents the overview of the problem; Section 4.2 shows how we propose to use DRL in the definition of the best emergency exit routes for large buildings in real time; Section 4.3 describes in detail our model and tool for representing the floor plan images in a graph; and Section 4.4 describes the settings for training.

### 4.1. Overview of the Problem and Solution

In summary, we define our approach as follows:

- Problem: Given a plan of an indoor building and a situation of danger of fire, it is intended to give quick and accurate answers to the building’s occupants.
- Goals: Find safe paths from different danger positions in the building to safe positions in the plan within a few seconds at the most, taking into account constraints imposed by the situation.
- Proposed solution: To achieve this goal, the following steps and tools were developed:
  1. A modeling tool, such that a plan can be defined graphically and transformed into a graph, where each room is indicated as a node, the edges are the possible paths (e.g., a door or window), and the safe node is indicated as the objective (at the end).
  2. Given the graph mentioned above, our solution uses DRL algorithms to find the best emergency exit routes in the shortest possible time and with the highest possible accuracy. For this, the problem is modeled using the pattern of OpenAI Gym and a DRL algorithm based on experiment replay and target network is also defined. Three alternatives are investigated: using random initialization, without random initialization, and to rely on transfer learning instead of initializing the repetition buffer. Once the best DRL strategy is chosen, the algorithm can be applied in real time.
- Outputs. Discovered safe paths to exit routes, which can be used, for instance, to give quick response to victims in danger situations and rescuers in real time through an APP.

The details of each step of the proposed solution are presented in the next subsections.

### 4.2. The Use of Deep Reinforcement Learning for Defining the Best Emergency Exit Routes

**Figure 1:**
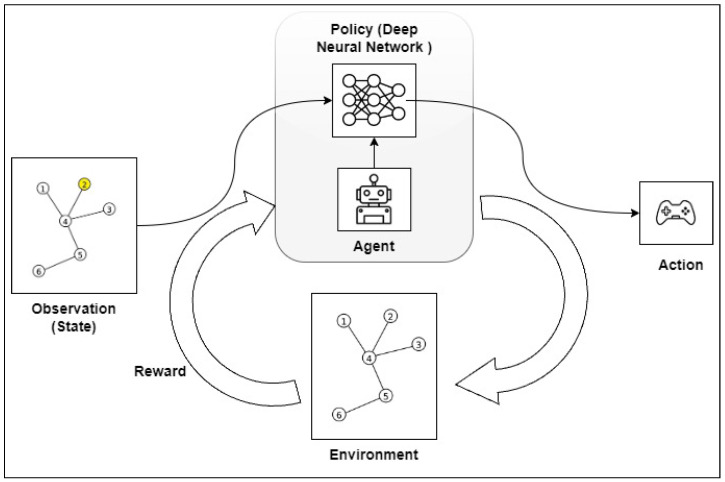
Pattern of OpenAI Gym.

A close scrutiny of the problem shows that it is feasible to use the Q-learning algorithm to find the best route from an initial node to a final node within a graph. However, finding the best route from all the nodes of the graph to the output nodes using a single Q-table becomes a challenging task. We recommend the use of DRL to avoid training a learning agent multiple times for different initial nodes and, hence, different paths. It is expected to be more efficient for addressing complex problems in real time. The  proposed algorithm follows the pattern shown in Figure 1.

As shown in Table 2, a fully connected neural network was implemented. The network architecture consists of four fully connected layers, each of which helps to change the dimensionality of the output of the previous layer. This model can easily define the relationship between the values of the data that the model is working with and the non-linear activation function called the rectified linear activation function (ReLU).

The planned neural network includes two input parameters: (1) the number of actions that the agent can carry out within the environment, i.e., estimating the number of nodes that exist in the graph; and (2) the size of the graph of the observed environment, i.e., for measuring the number of nodes × 2. Thus, the agent will have a partial observation space in the environment, since the environment used is a matrix that has a size:

(5) $$\mathrm{number}\;\mathrm{of}\;{\mathrm{nodes}}^{2}.$$

The planned solution entails the using the adaptive moment estimation (ADAM) algorithm, as it an is efficient tool for handling large problems involving many data or parameters. Moreover, it does not require too much memory. As an extension of a stochastic gradient descent method, the ADAM algorithm is used to interactively update the network in accordance with the training data.

The MSE method was chosen to calculate the loss and is widely used in machine learning problems to check if the models are correct, as it attributes greater weights to the largest errors. In the MSE, the errors are squared individually and then the average is obtained between the calculated errors.

The output of the network includes an array of possible actions. This array contains a value linked to each possible action that must be taken within the environment. The best action to be taken is the one with the highest value.

The OpenAI Gym [32] was used for the implementation of the DRL algorithm. This is one of the most popular reinforcement learning libraries. Its structure facilitates the understanding of the problem and enables future changes to be made, as it adheres to a well-established pattern.

Our solution employs three methods of the environment class (from OpenAI Gym) and can be described as follows:

- The init method is used to initialize the variables necessary for the environment. A graph referring to the floor plan is provided with information. Following this, an adjacent matrix is created from the input graph, output nodes, and the nodes affected by fire. The spatial distance between the nodes is used as the edge weight. The number of possible actions to be performed is defined in terms of the number of nodes in the graph and the size of the observation space is defined as the number of possible actions × 2.
- The reset method is used to reset the environment to its initial conditions. This method is responsible for defining the initial state of the agent in the environment, either in a pre-defined node or randomly, if it has not received the information. By using random initial states during the training, the agent learns the path from all the nodes of the graph to the output node, although this also makes the training time much longer.
- The step method is responsible for the core of the algorithm, as it uses the neural network to define the next action required, the next state, and the reward. It receives the current state and the action as parameters. After this, it calculates the reward by means of the adjacent matrix, which shows the adjacency and distance between the nodes.

The values of the returned rewards are defined as follows:

- If the next state is a node that is on the list of nodes affected by fire, the agent incurs a severe penalty (i.e., a negative reward) of −10,000.
- If the next node is not adjacent to the current node (i.e., they are not neighbors), the agent incurs a penalty (negative reward) of −5000.
- If the next node is adjacent to the current node (i.e., they are neighbors) and is not an exit node, the agent incurs a penalty (negative reward) corresponding to the weight of the edge that connects these nodes.
- If the next node is an exit node, the learning agent is granted a positive reward of 10,000, which is a sign that it has successfully completed its goal.

After the graph in the web interface has been drawn, all the node coordinates are known. This means, the Pythagorean theorem can be used to calculate the distance between these nodes. According to this theorem, if there is a node *A* and a node *B*, where $A(x_{a},y_{a})$ and $B(x_{b},y_{b})$, the distance between these nodes can be calculated using Equation (6). This distance is used to calculate the edge weights and the agent’s reward, and hence, determine the agent’s reward in the third case described above.

(6) $$d_{AB}=\sqrt{{\left(x_{b}-x_{a}\right)}^{2}+{\left(y_{b}-y_{a}\right)}^{2}}.$$

The agent class is responsible for initializing the planned neural network in the environment and orchestrating the training. The parameters required to initialize the agent include the graph with the floor plan, the exit nodes, the nodes affected by fire, and the decision on whether the transfer learning method should be used. If transfer learning is used, it is also necessary to provide a weight parameter. It is also necessary to initialize the variables used in the training, as well as employing experience replay variables and target network techniques, such as the replay buffer.

The replay buffer is a part of the experience replay technique, where the experience is represented by a quadruple consisting of the current state, action, reward, and new state. This buffer can be initialized with random values of transitions before the agent training. Thus, at this stage, random values are used to choose the actions that need to be carried out. This random initialization seeks to help in the training of the agent, so that it can start the training with some previous knowledge. If you decide to initialize this repetition buffer before training, it is necessary to define how many early experiences will be previously collected (e.g., 25% of the total buffer size).

**Figure 2:**
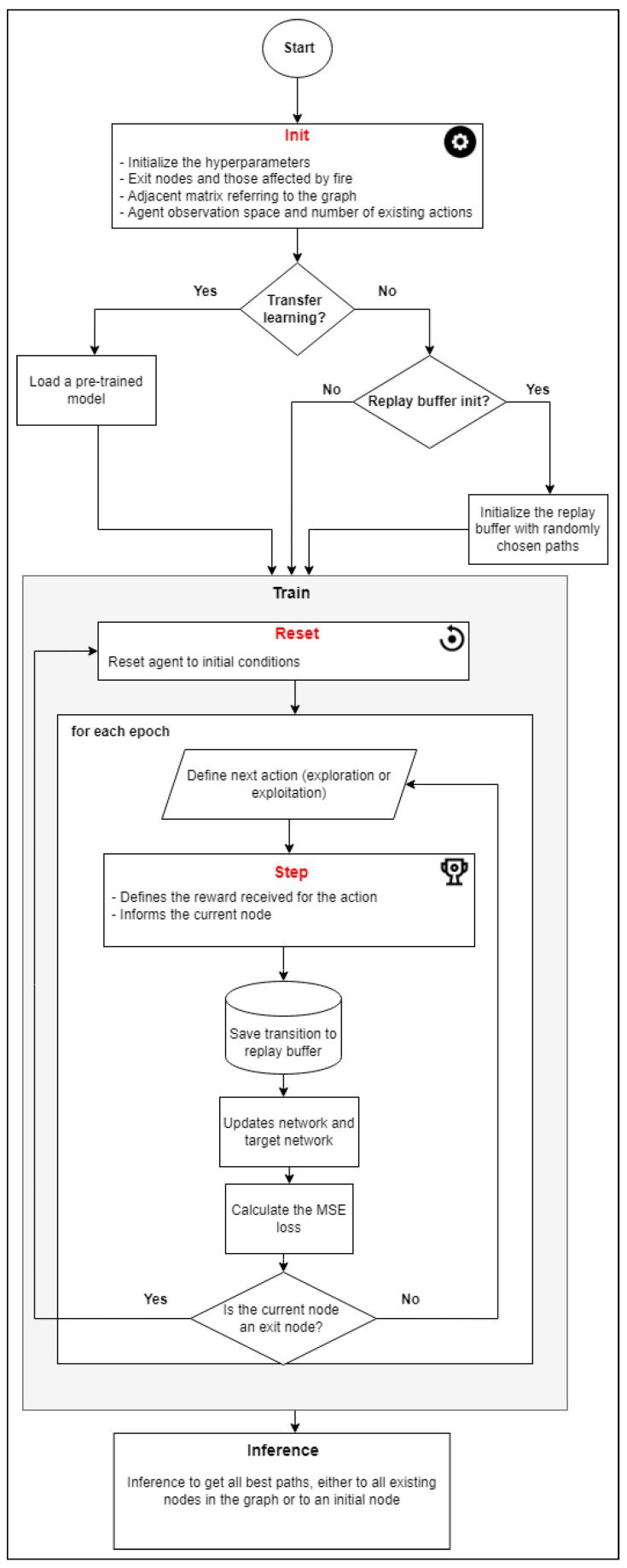
Deep reinforcement learning algorithm flow.

The flow of the algorithm is visualized in Figure 2, from which it is possible to understand the operation of the training algorithm, as well as the relationship and importance of each of the methods discussed here.

Algorithm 1 defines an agent-training algorithm with the experience replay and target networks adopted in this study. This algorithm shows the process followed in each training episode. The first part is where the defined concepts of exploration and intensification will be used; the exploration rate will be defined as *epsilon (ϵ)* and will be responsible for balancing the two techniques inside the algorithm.

The definition of *epsilon (ϵ)* is shown in Equation (7), which means that, for the first few episodes, the probability of choosing an action by the exploration method is very high, since the action will be performed randomly, but this declines as the episodes occur, since the intensification method will be given priority and the action will be carried out on the basis of the neural network.

(7) $$\varepsilon =exp(\frac{-i}{\frac{\mathrm{number}\;\mathrm{episodes}}{2}}).$$

The second part of Algorithm 1 refers to the experience replay and the target network. It is in this part that the batch size variable will be used, and this will indicate the size of the mini batch of random samples from the repetition buffer, which will be used to update the neural network. The *gamma (γ)* variable is also used for calculating the targets in the target network. In theory, the network should not be learned until there is a pre-defined value for the size of the repetition buffer, but since the algorithm has to operate as quickly as possible, the network learning stage takes place in all the episodes.    
**Algorithm 1:** DRL algorithm based on experiment replay and target network
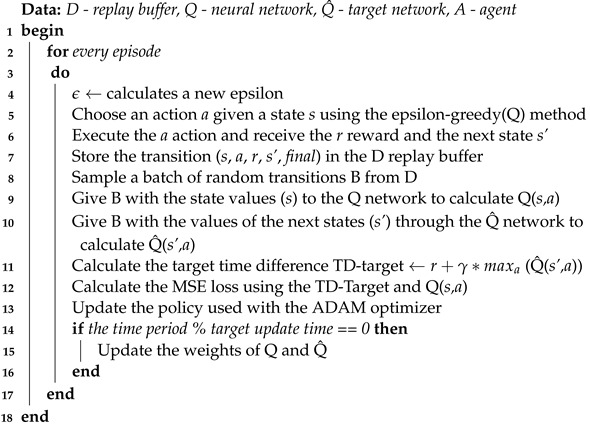


The inference time represents how long a deep learning model takes to make predictions on new data. This is when the model uses what it learned during training to make these predictions, a process known as inference. It is important to note that this time is different from the training time of the model. This project will show both how long it takes to train the agent and how quickly it can make predictions.

### 4.3. Representing and Using Floor Plan Images

As the main objective of this work is to conduct an analysis of escape routes using RL, a suitable representation, such as graphs and matrices, is essential. However, the question of providing an automated representation is an open research topic, and is not the focal point of this work. In light of this, we decided to seek a representation with greater accuracy and reliability by following a manually assisted process. A web application was created to this end.

The developed application was constructed by means of a Vue Javascript framework [33] and the FastAPI, a framework for developing RESTful applications in Python [34]. The MongoDB, a document-oriented database, provided the data persistence solution [35].

**Figure 3:**
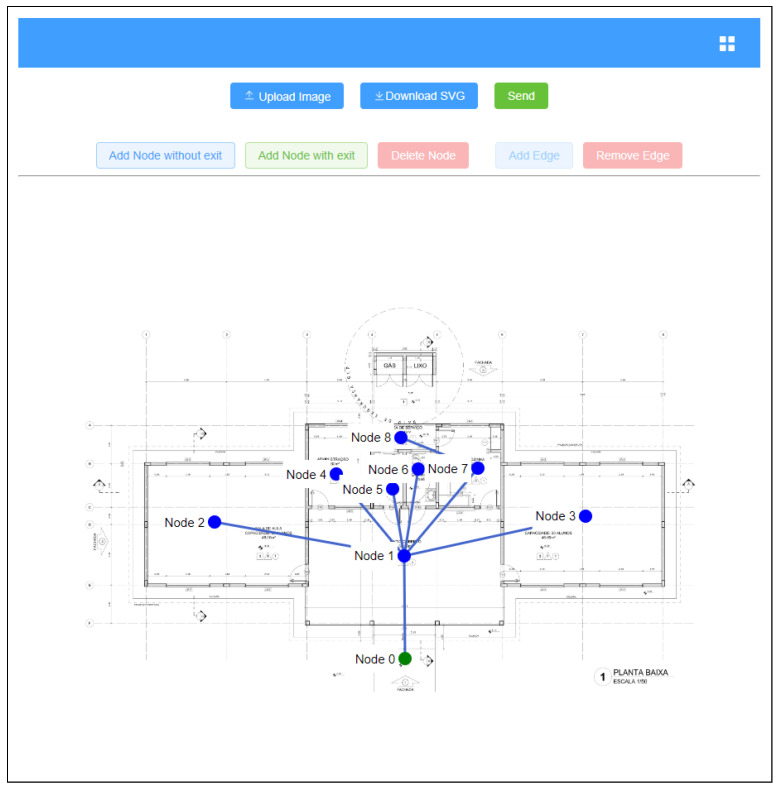
Creating a graph using a floor plan.

First of all, a user can include an image of a floor plan of the building (JPG, JPEG, or PNG) in a project. Then, the user can draw the nodes and edges corresponding to the rooms and connections between them on the image that has been sent. As Figure 3 shows, a graph is created from these drawings. The v-network-graph [36] library was used in the implementation of this feature.

Nodes with no exits are marked in blue, which indicates that there is no exit from the building in the represented room. Nodes with exits are shown in green, which indicates that there is an exit from the building in that room. The edges mean that the two nodes have a direct connection, through doors, doorways, or corridors, i.e., they are adjacent nodes.

Some factors should be considered when creating the graph for the application, the most important of which are as follows. If it is uncertain whether a window can be used as a passage from one location to another, they should not be regarded as alternative paths. The node most be located as closely as possible to the center of the room, since its coordinates are used to calculate the distance between adjacent nodes. It is essential to ensure that all the information contained in the graph is correct, such as the number of nodes, edges, and the nodes that have a safe exit from the indoor environment.

The features of the project, including the image and graph, are stored in a MongoDB database. This allows them to be retrieved and changed as needed by the user. This makes it a more secure and reliable persistence solution than a simple file storage system, as well as speeding up the procedures for retrieval, use, sharing, and update by users. We also developed a set of RESTful web services which are responsible for the interaction between the web interface and the database. The python FastAPI [34] was used to this end.

In the design time, for the inference (execution) of a current project, it is necessary to query the database, select the project, adjust the execution parameters and, finally, execute the inference. The execution parameters include the RL model, the set of nodes affected by fire, and responding to the need for (re)training a specific node. (Re)training a specific node can be achieved faster than (re)training all the nodes. In real-time execution, the project, training, and parameters are previously defined or given information by the sensors (e.g., the nodes affected by fire).

Thus, it should be noted, that before inferring a project in real time, its information must already be previously stored in the database, since the process of creating the graph that represents the floor plan is a manual task, which needs a lot of attention and in some cases is time consuming.

### 4.4. Settings for Training

The purpose of carrying out experiments is to make an assessment of the proposed DRL-based solution in three environments, with different numbers of nodes and edges. These environments are used to measure the effects of the solution in the analysis of emergency exit routes.

Our analysis is focused on the floor plans of public schools. Schools were chosen because they are critical environments in the event of fire and pose serious challenges, such as the following: the fact that many people are crowded into an indoor environment, the need for a quick response, and the fact that they have more complex and larger plans than houses or small offices. Furthermore, they are all real buildings and easy to compare throughout the experiment. These floor plans were made available by the National Fund for Educational Development, a federal agency under the auspices of the Brazilian Ministry of Education [37].

The number of training episodes is defined in accordance with the floor plan under analysis. In our experiment, the number of episodes is shown in Table 3. A key factor is the number of edges, since as the number of possible paths to be taken by the learning agent grows, the problem will become more complex. One of the alternatives that reduces the need for a large number of episodes is the use of the transfer learning technique, as it uses pre-trained weights, which facilitate the agent’s learning.

Three RL alternatives were deployed, all using experience replay, but with a different initialization strategy to initialize the repetition buffer: (1) with random values, (2) without random initialization, and (3) relying on transfer learning instead of initializing the repetition buffer. For this reason, it was decided to divide all the experiments into three stages.

In addition to analyzing the best paths recommended by the algorithm, two metrics were used for evaluating the training: the average reward accumulated over the episodes and the MSE loss.

In assessing the need for (low) computational resources, all the experiments described in this work were carried out on a machine with an AMD Ryzen 5 3500u processor, with an integrated NVIDIA GeForce MX250 video card and 8 GB DDR4 RAM memory at 2400 MHz.

## 5. Projects, Results, and Discussion

This section describes the experiments, presents the results, and provides discussion of the three selected projects that address school floor plans. Each project has a different plan size and evaluates the performance of the methods in sequence. The three proposed methods, experience replay with buffer initialization, experience replay without buffer initialization, and transfer learning are compared in this section. This section is divided into five parts: Section 5.1, Section 5.2 and Section 5.3 detail projects 1, 2, and 3, respectively; Section 5.4 presents a summary of the results obtained from all the experiments, while Section 5.5 discusses potential system improvements and challenges.

### 5.1. Project 1

Project 1 refers to a floor plan for rural schools with an intake up to 120 students. It contains only two classrooms, and this plan is used for the construction of schools in small rural communities in several regions in Brazil.

**Figure 4:**
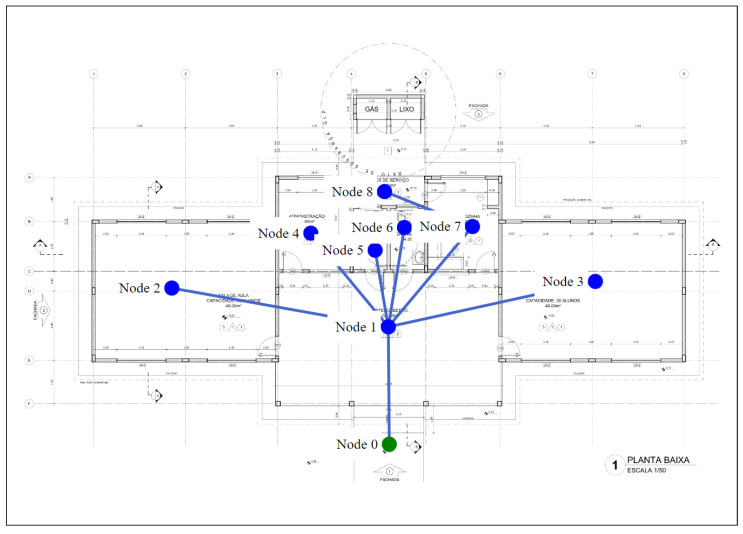
Floor plan and graph of project 1.

As shown in Figure 4, project 1 has nine nodes and eight bi-directional edges, and only one output, i.e., node 0 is the only one that is green. This design can be considered to be simple, since with the exception of node 8, all the nodes have short paths to the output node. The training settings for project 1 are displayed in Table 4.

In the first simulation scenario, there is no room in the building affected by fire, so there are no blocks between the graph nodes and the output node. The aim was to analyze the agent training time for the agent and the recommended methods to find the path from all the nodes in the graph to the output node. The initial node was not shown in this scenario, and thus, the paths of all the nodes to the output node can be found.

The DRL method was used, together with the experience replay technique that had 4000 episodes. The agent’s training time when the repetition buffer was not initialized was 33 s. In cases involving training with random initialization of the repeating buffer, the initialization training time was 0.7 s, while the training time for the learning agent was 39 s.

**Figure 5:**
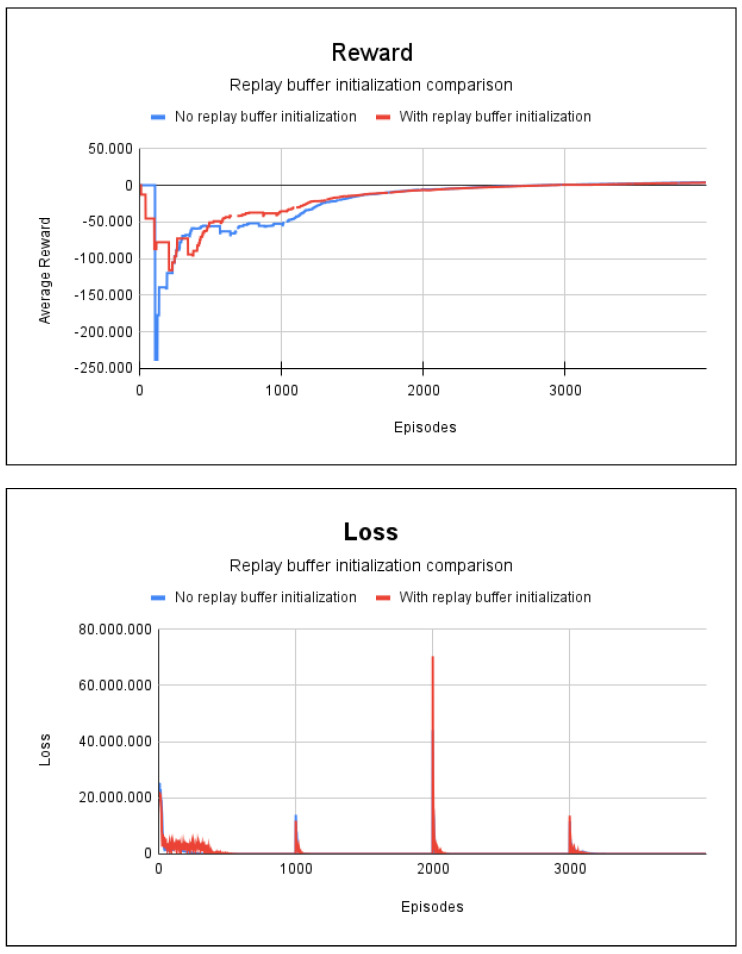
Reward and loss charts for project 1: no blockages/fire scenario.

The results for the accumulated reward and the results of the loss for project 1 are displayed in Figure 5. From an analysis of the results, it can be seen that there is no substantial difference between initialization with random and non-random repetition buffer values; since in both cases, the accumulated reward begins to converge from episode 2500. Thus, when there are small graphs, the only significant difference between these two methods is in the inference time, that is, the strategy that did not employ random initialization values for the buffer is a little faster. In these cases, 100% of all the possible paths in the environment were found.

The path of node 8 to the existing node is the only one with more than three edges (node 8, node 7, node 1, node 0). In the cases of the other *n* nodes, there is a path through node 0, i.e., ${\mathrm{node}}_{n}$, node 1, node 0. Thus, if a fire occurs at node 1 or node 7, there will be no safe paths to the output for one or more nodes in the graph. In light of this, a simulation scenario was created to find out what the agent would learn if node 7 was blocked. Blocking node 7 results in safe paths from node 8 being impossible, so we evaluated a scenario with this situation.

The transfer learning method was used in a scenario with fire in node 7. It should be noted that the weights obtained in the previous scenario, without fire, were used, instead of the random initialization of the repetition buffer. This means, the agent starts by using a pre-trained weight which resembles the current scenario, except for the fact that node 7 is blocked.

As expected, the agent was not able to learn the paths from node 8 in the scenario with fire in node 7, although it was able to learn the safe exit paths from the other nodes. As a result, 86% of the total number of paths and 100% of the safe paths in the environment were found. It is worth noting that only 1400 episodes were used for the transfer learning method, which had a training time of 12 s. This is half the time expended to train the agent without pre-trained weights.

**Figure 6:**
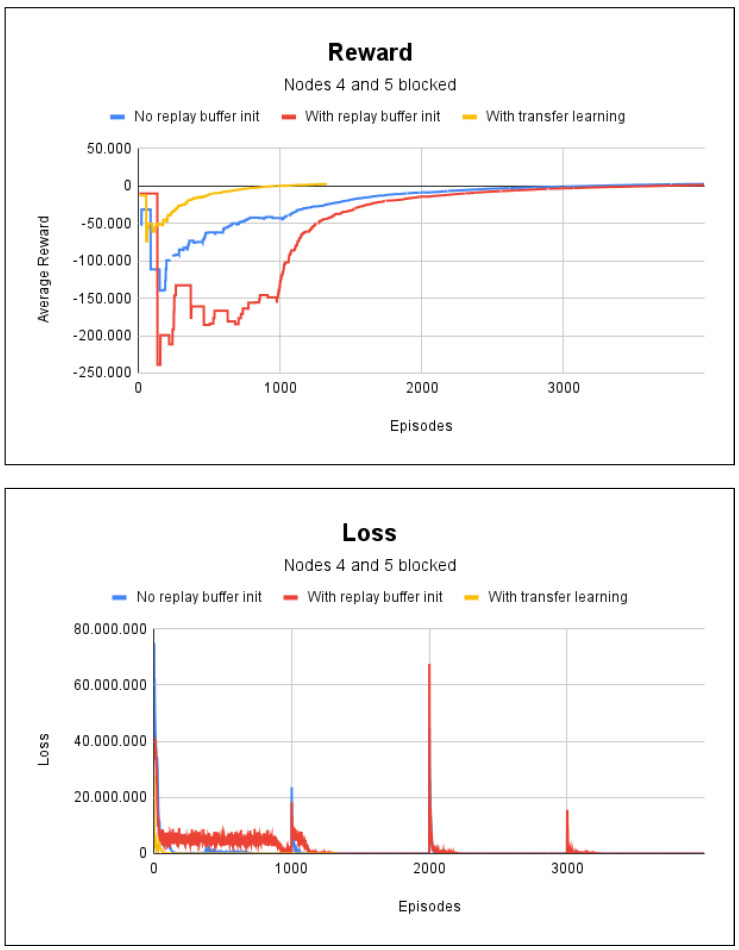
Reward and loss charts for project 1: with fire at nodes 4 and 5.

In the last scenario, we simulated fire in nodes 4 and 5. The agent took 51 s to train for the experience replay technique without random initialization of the replay buffer; with the random initialization of the replay buffer, it took 1 min. In both methods, 4000 episodes were necessary. Only 1500 episodes were required for the experience replay with the transfer learning case. This resulted in a training time of 16 s. The averages for the accumulated reward and loss for this experiment are shown in Figure 6, where in all cases, an accuracy rate of 100% was obtained in the environment paths.

Figure 6 shows that transfer learning had better results, as it required fewer episodes for training the agent, which speeded up the training. With regard to the scenarios shown in Figure 5 and Figure 6, we did not find any significant difference in the training time due to the presence of fire, since the learning of agents begins to converge after episode 2500.

### 5.2. Project 2

Project 2 refers to a rural educational space with four classrooms and a sports court. This plan is used for the construction of schools in various regions in Brazil. These schools have a capacity of 240 students (i.e., twice the capacity of project 1).

**Figure 7:**
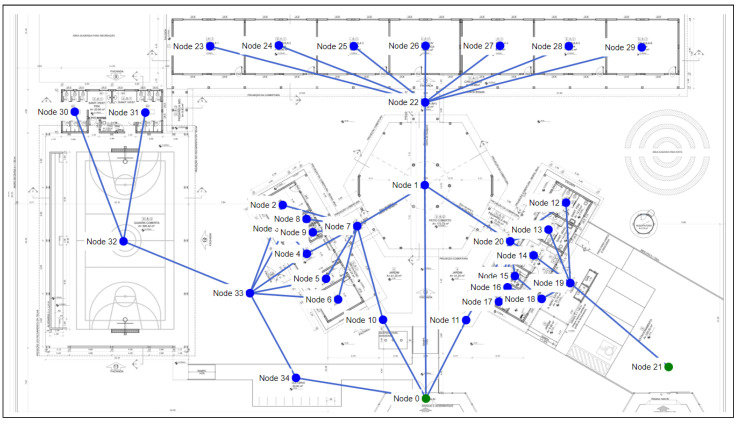
Floor plan and graph of project 2.

As shown in Figure 7, project 2 has 31 nodes and 34 bi-directional edges. This plan has two outputs, which are represented in the graph by node 0 and node 19 in green. Project 2 has greater difficulties than project 1, as it has a significantly larger number of nodes and edges. Owing to its construction features, the windows cannot be considered to be a passage. The training settings for project 1 are in Table 5.

In the first simulation scenario for our experiment with project 2, there is no room/node affected by fire. Thus, there is no blockage between all the graph nodes and the output nodes. The purpose of this scenario is to analyze the agent’s training time with a view to finding the path from all the nodes in the graph to the output nodes.

**Figure 8:**
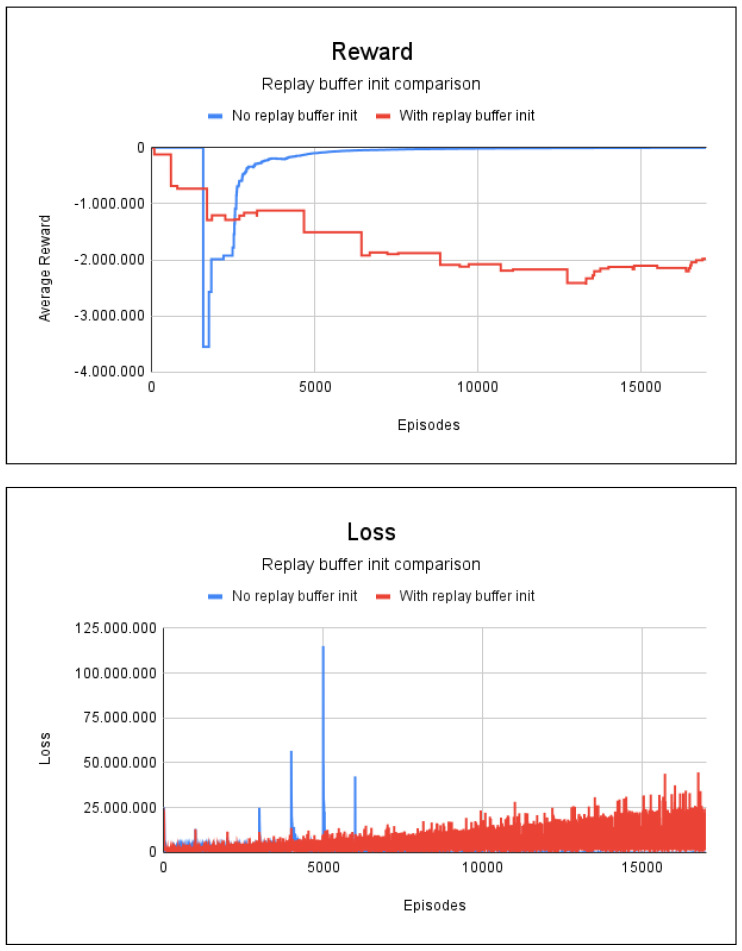
Reward and loss charts for project 2: no blockages/fire scenario.

The experience replay method without the replay buffer and with random values took 2 min and 53 s for training. In the case of initialization of the replay buffer with random values, the time for the initialization of the buffer was 0.6 s, the training lasted 2 min and 39 s. Both methods required 17,000 episodes. Figure 8 shows the graphs for the average accumulated rewards and losses.

Figure 8 shows that the random initialization of the repetition buffer can end up harming the agent’s learning, because even after 17,000 episodes the agent managed to learn only one path within the entire graph, with a degree of accuracy of only 3.26%. This resulted in a very low path accuracy rate. Without random initialization, it only took 7000 episodes for the agent to learn all the safe paths, with an accuracy rate of 100%.

As in project 1, we analyze the effects on the agent if there are blocked (fire) nodes within the environment of project 2. In the second scenario of project 2, we blocked node 2, which meant there were no safe paths from nodes 3, 4, 5, 6, 7, 8, and 9 to the exits in this scenario. The method without transfer learning required 17,000 episodes and the method with transfer learning 5666. Unlike the results obtained in project 1, none of the methods was able to find the safe paths from the graph nodes to the output nodes, and thus, the path accuracy rate within the environment was 0%. The reason for this was that in the case of many nodes, the reward offered was extremely low, regardless of the path chosen, and this harmed the agent’s learning. In view of this, we suggest that the paths predicted by the algorithm should not be included and, if necessary, to search for the path of only one node can be used at a time.

A third scenario simulates fire at nodes 11, 12, 13, 14, 15, 16, and 17. Again, 17,000 episodes were used for the methods without transfer learning and 5666 for the method with transfer learning. It took 2 min and 40 s to train the agent without any random initialization of the replay buffer. With the aid of random initialization, it took 0.6 s to initialize the buffer and 2 min and 38 s to train the agent. By using transfer learning, it took only 52 s to train the agent, which is less than half of the time needed with other methods.

**Figure 9:**
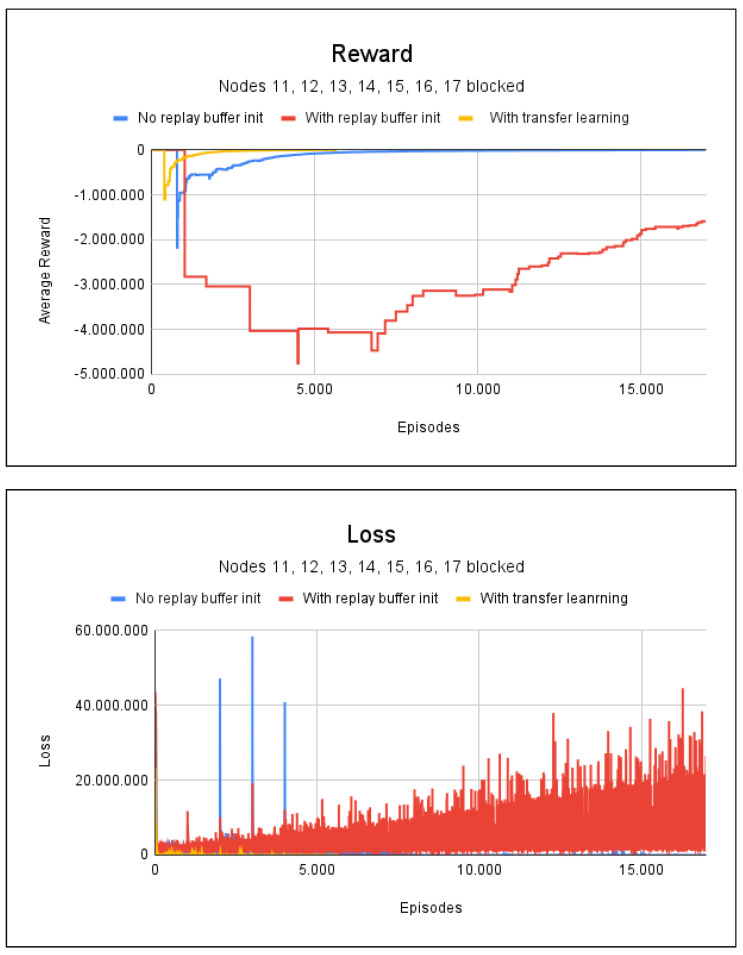
Reward and loss charts for project 2: with fire at nodes 11, 12, 13, 14, 15, 16, and 17.

However, from an analysis of Figure 9, which shows the average accumulated reward and loss, it can be seen that the technique of initializing the buffer with random values still returns bad and unwanted results, with a path accuracy rate of only 7%. Again, the technique of using transfer learning stands out, as only 4000 episodes was needed for convergence, with a training time of less than 1 min and a path accuracy of 100%.

The results show that initialization of the repetition buffer with random values was not a viable alternative for the studied project. Thus, we focused on understanding better how the transfer learning technique can be used in RL.

### 5.3. Project 3

Project 3 refers to a floor plan of a rural educational space with six classrooms and a sports court. This plan is used for the construction of schools in various regions in Brazil. These schools have an intake of 360 students (i.e., three times the intake of project 1).

**Figure 10:**
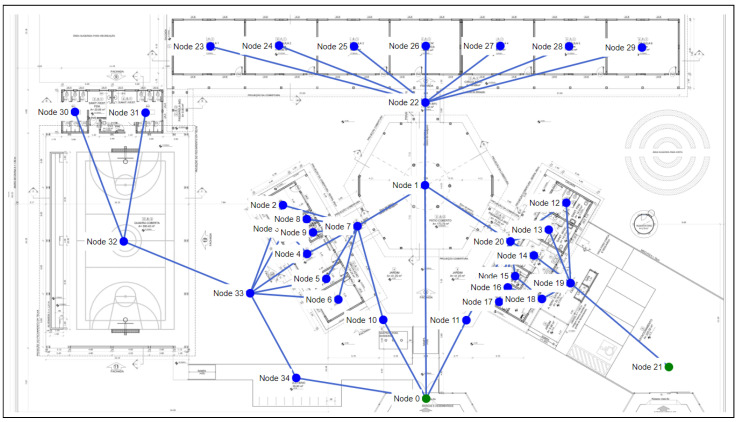
Floor plan and graph of project 3.

As shown in Figure 10, project 3 has 34 nodes and 46 edges. Some windows can be regarded as passages to the exit in the project, resulting in a considerable increase in the number of edges in the graph when compared with project 2. Node 0 and node 21 are the output nodes of the graph. The training settings for project 1 are shown in Table 6.

In the first simulation scenario for our experiment with project 3, there is no room/node affected by fire. Thus, there is no blockage between all the graph nodes and the output nodes. The purpose of this scenario is to analyze the agent’s training time to find the path from all the nodes in the graph to the output nodes.

The agent took 41,400 episodes for the paths with a 100% degree of accuracy with the method without random initialization of the replay buffer, but without transfer learning. The duration of training was 7 min and 14 s. Thus, there was a larger increase in the required time than with project 2. Since the graph in project 3 has two nodes and 14 more edges, it is clear that an increase in the number of edges and nodes greatly influences the agent’s training time, as it is necessary to choose large numbers of episodes.

As in the previous projects, we used the weights obtained in the first scenario to evaluate the use of transfer learning in the others. In the second scenario, node 32 was blocked (fire), and as a result, there are no safe paths from nodes 30 and 31. The agent used 41,400 episodes without using transfer learning, but despite this, it failed to find any safe path for learning within the graph. While using the transfer learning method, only 7666 episodes and 1 min of training time were required. This method was also successful in obtaining 90% of the safe paths, and thus, achieved the best performance from the transfer learning method.

The transfer learning method also had a superior performance when node 22 was blocked (fire). This method obtained a 90% accuracy rate for finding safe paths, while the method without transfer learning did not find any paths (i.e., 0% accuracy).

As mentioned above, in some cases the solution was not able to achieve 100% accuracy in safe paths, even with the use of the transfer learning method. Thus, we tried other strategies to improve it, including the indication of an initial node, as shown in the following paragraph.

**Figure 11:**
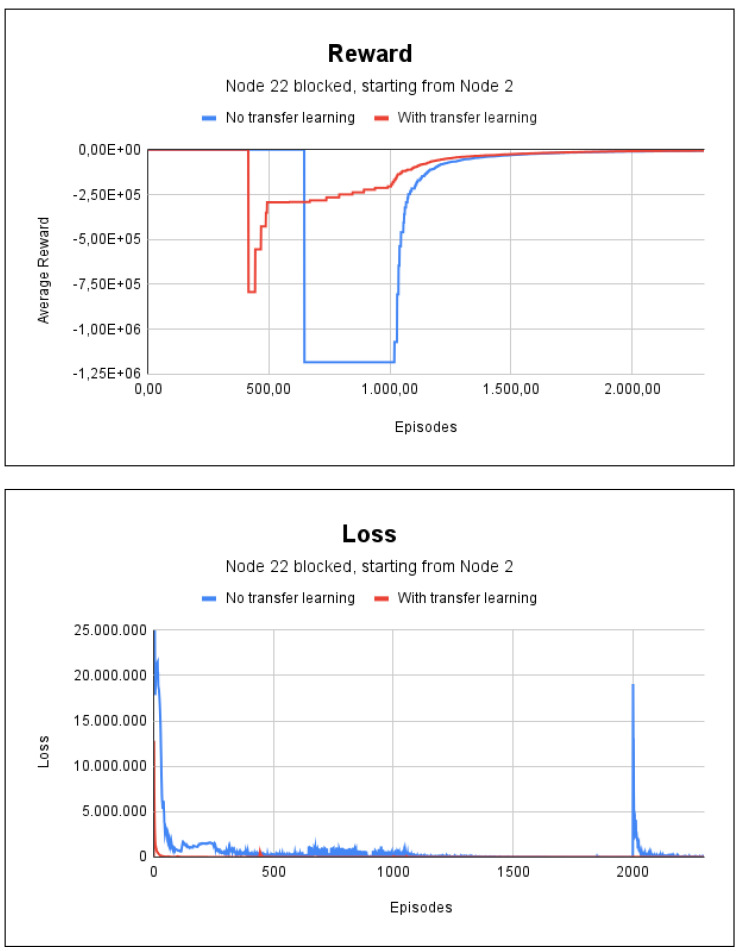
Reward and loss charts for project 3: indicating node 2 as the initial node and fire at node 22.

We indicated node 2 as the initial node in the scenario with fire for node 22, which is one of the paths where the method failed in previous tests. In this case, the method adapted quickly and accurately found the path. The training lasted 21 s without the use of transfer learning and lasted 20 s with transfer learning. As shown in Figure 11, only 2300 episodes were needed in both methods. The reason for this was that the pre-trained model was designed by using all the nodes of the graph, i.e., not for a specific one.

**Figure 12:**
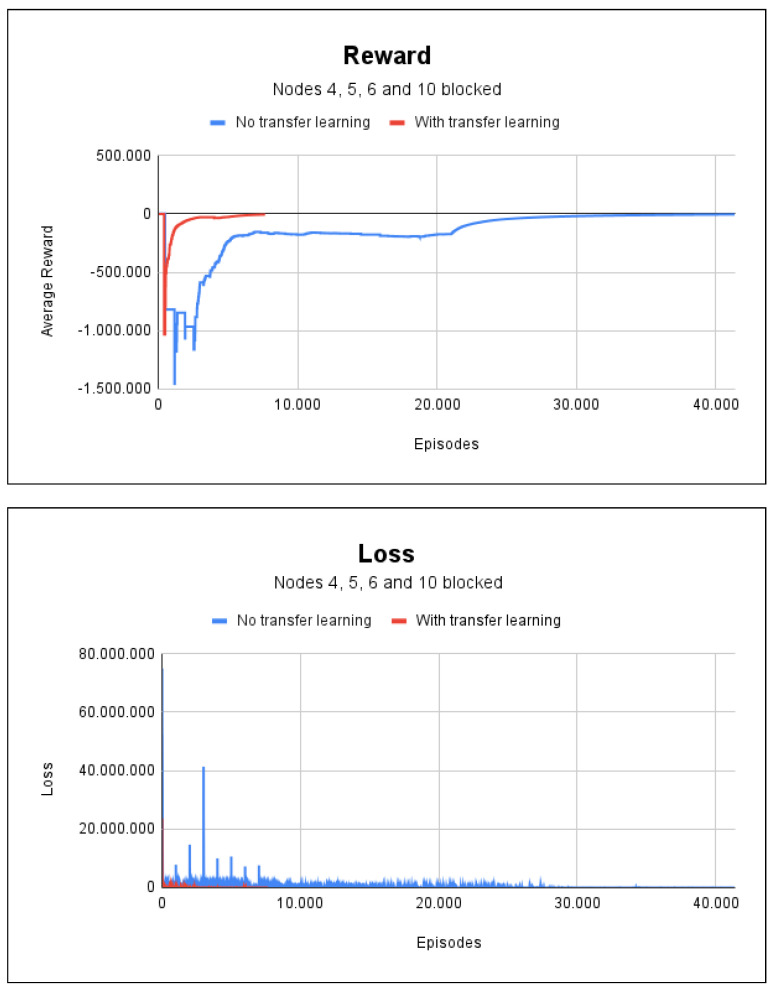
Reward and loss charts for project 3: with fire at nodes 4, 5, 6, and 10.

Finally, the effectiveness of having several blocked nodes in project 3 was determined by carrying out a simulation scenario with fire for nodes 4, 5, 6, and 10. The training without transfer learning needed 41,400 episodes and took 10 min and 21 s. While the training with transfer learning needed 7666 episodes and took 1 min and 48 s. Figure 12 shows the accumulated rewards and losses for this scenario, and makes clear that transfer learning accelerated the training of agents. Both methods achieved a 100% degree of accuracy in this scenario.

### 5.4. Summary of Projects

A summary of project 1 is presented in Table 7, a summary of project 2 is presented in Table 8, and a summary of project 3 is presented in Table 9. These tables present the results for accuracy, training time, and inference time obtained in each of the simulations.

The average inference times calculated for the project 1 experiments, project 2 experiments, and project 3 experiments were 0.035 s, 0.56 s, and 0.21 s, respectively. Analyzing the results reveals a pattern of increasing inference time as the environment size grows. Another intriguing observation is that the inference time tends to be higher when the agent struggles to determine safe pathways to the exit, resulting in lower accuracy. This might explain the higher average inference time in project 2 experiments, where five out of eight trials showed reduced accuracy. In contrast, in project 3, only two out of seven experiments displayed a lower accuracy rate.

### 5.5. Improvements of the System, Discussion, and Challenges

In this subsection, we outline and discuss the improvements made to our system, based on the results obtained in the experiments, as well as to discuss the results and open challenges that need to be addressed in this research study.

#### 5.5.1. Improvements in the System

**Figure 13:**
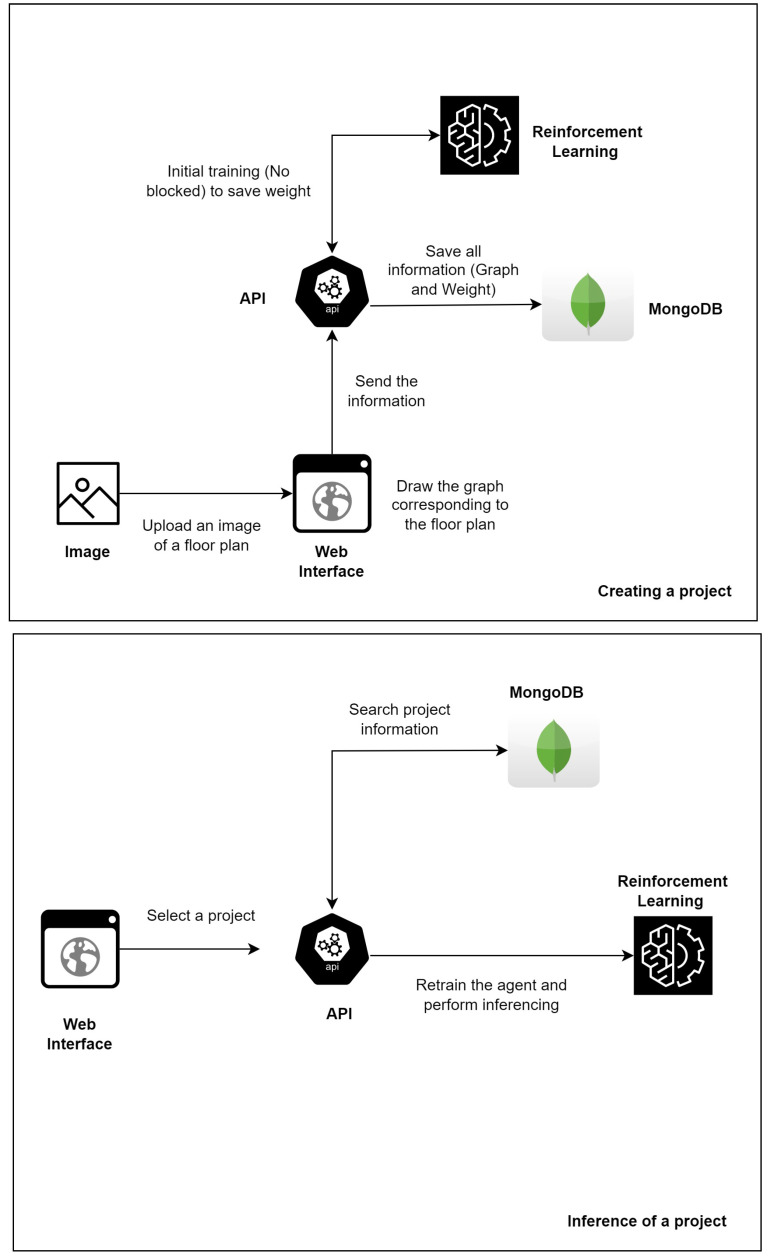
Improved solution architecture overview.

Figure 13 provides an overview of the improved method after the obtained results have been analyzed. It includes the functionalities which are designed to quickly select reinforcement learning alternatives (a) to avoid the problem of being unable to find safe paths (e.g., the choice of initial nodes), (b) to make use of transfer leaning (which proved to be effective), and (c) to integrate the system with smoke sensors in the environment, for real-time monitoring.

On the basis of our experiments, RL with experience replay and transfer learning obtained superior results in most cases and the same results in the worst cases. Thus, when the user adds (or updates) a project through the web interface, the improved solution trains the agent by basing it on the information in the graph, without any blocking, so that the weight of this training can be used in the future. Thus, when the user decides to infer an existing project, training with transfer learning is immediately made available.

Integration with internet-connected smoke sensors is also desirable for a real-time response. The architecture of the improved solution provides components for retraining the agent when there are new blocks/fire (or the blocks are removed), which means the information about the safe paths for users is often updated.

#### 5.5.2. Discussion

Despite advances made in standardization and fire prevention technology, a rapid response systems to fire incidences is necessary to prevent thousands of injuries and fatalities every year. Quick and precise information about safe emergency exit routes for occupants and firefighters is a key factor in such a dangerous situation. Owing to limitations in the performance of well-known path algorithms for real-time problems [10,38], in this article we propose the use of RL to provide the rapid evacuation of occupants from fire outbreaks in indoor environments.

Our results show the feasibility of using RL for this purpose in different scenarios of fire in three school projects. These results are in line with other previous studies that require real-time path solutions, such as games and transportation [39,40]. In the following section, we discuss the individual results of each project, followed by a general discussion, as well as noting the limitations of our study and future challenges that have to be faced.

Project 1 is the simplest one, as it includes a graph with few nodes and few edges. In a situation without blocked nodes (fire), we obtained very similar results for both cases, both with and without random initialization of the repetition buffer. This is due to the relative simplicity of the problem, where there are few paths that the agent can take from a specific node, and thus, initializing the repetition buffer with random values before training will not cause any bias in the agent. At the same time, it does not lead to any difference or improvement when compared with a situation where the buffer is not initialized with these values. However, when there are blocked nodes (fire), it is possible to use pre-trained weights (i.e., previously trained without blocking). In this scenario, the transfer learning stands out from the others, and only 1500 episodes and 16 s are required to return safe paths with high precision. This is because the weight refers to training that is carried out to find all the existing paths within the graph, and now it will only be necessary to retrain the learning agent for some paths that start from specific nodes.

Project 2 shows that the random initialization of the repetition buffer was ineffective with a greater number of nodes and edges. Random initialization can hamper the learning process, because there is no control over the transitions or the use of samples from the repetition buffer to update the neural network. Without this control, bad transitions might be chosen, that can confuse the agent and hinder learning. In view of this, the random initialization of the repetition buffer proved to be ineffective for the RL process of our agent. Again, transfer learning was the most effective and fastest method.

Project 3 shows that the transfer learning technique is the fastest and most effective of the large-scale building plans, since it requires only 1 min and 28 s to accurately return all the paths from the graph nodes to the output nodes.

The transfer learning method is effective because it transfers the weights of the network that learned the task, i.e., finding all the paths of all the existing nodes in the graph, which leads to a new task where there is blocking of some nodes, although it is similar to the previously learned one. This makes training more efficient, especially when the training time is taken into account.

Training with blocks is faster than when it is carried out without blocks and the user creates the project via the web interface; this is because training with blocks benefits from pre-trained weights (the transfer learning method). As a result, in emergency situations, the best routes can be calculated from all the rooms in a building to the exit in a matter of seconds or minutes.

As mentioned above, this performance cannot be achieved by well-known path algorithms such as Dijkstra, Bellman–Ford, and A* [10]. With the aid of DRL, the best path can be found from all the nodes of the graph to the output node. This would be difficult to achieve with traditional path search algorithms, as they do not perform well when there are multi-objective problems [11], since they have to make the calculation of the best path for each node separately, which incurs computational costs and a longer time to solve the problem [41].

Our experiments also point out that the algorithm may fail in cases where an essential node is affected by fire. If a node is blocked by fire, this interferes with the safe paths from one or more nodes within the graph to the output. In addition, the training of the learning agent may be unsatisfactory, even with the use of the transfer learning technique. However, these cases can be avoided by training the agent with an initial node. By using an initial node within the graph, learning is fast and accurate, as the agent will only have to learn one path.

Thus, by combining all the components examined in this study, it was possible to form a final application, called EvacuAI, that is simple to use, making it easy to find several paths within a graph in real time.

The evaluation carried out in this article is limited in terms of the size of the plans tested. Additional studies are needed to evaluate the application with huge plans; however, it should be noted that the experiments were carried out with the aid of a notebook with a low-end configuration, which is not ideal for ML training. This made it possible to assess the feasibility of using RL in real situations if complex computational infrastructures are not available. Significantly better ’time performance’ results can be achieved with a high-performance ML infrastructure.

Another limitation of this work concerns not providing direct (numerical) performance comparisons with well-known algorithms; however, there are other differences that make RL algorithms more suitable for this study. Firstly, while well-known algorithms rely on exact mathematical knowledge, reinforcement learning constantly seeks the optimal policy through continuous optimization, making it more flexible and capable of dealing with imprecise environments in a more adaptable manner.

Our results are promising; however, long-term studies on real and simulated situations with people in danger, are needed before putting the system into production. These kinds of studies are costly, and require controlled environments as well as approval from authorities. There may also be technical difficulties, problems over communication, legal questions involved, and financial costs. Research on human behavior in situations where there are a large number of occupants, and consequently generating the best decision based on this, should also be studied in the long term. These factors must be addressed by researchers from various disciplines, representatives of companies, and public policy-makers.

## 6. Conclusions and Recommendations for Future Work

ML is currently widely used and it is constantly evolving; although RL is still under-explored, especially in real-time situations. This is particularly difficult because there is a need to retrain the learning agent if there are any changes in the learning environment.

This paper investigates the use of RL to find safe emergency exit routes in the event of fire in real-time. The floor plans of buildings were represented as graphs to this end.

We developed an interactive web application to facilitate the design of graphs from the floor plans. The projects, graphs, and annotations were stored in a database. An RL algorithm based on neural networks was created with the aim of finding safe paths from all the nodes of the graph.

We selected three projects to evaluate the experiments that were carried out to find emergency exit routes in public school floor plans in different scenarios. In these experiments, three alternatives systems were compared: (1) RL with experience replay and random initialization of the replay buffer, (2) RL with experience replay without random initialization of the replay buffer, and (3) RL with experience replay based on transfer learning.

The random initialization of the repetition buffer obtained the worst results, since it was not feasible in complex situations. The solution without random initialization of the replay buffer obtained worse results than transfer learning for the studied cases. The results confirm the viability of the transfer learning solution, as it provides an accurate and fast response in real time and requires few computational resources. Our studies also reveal the challenges that must be overcome so that our ’solution’ can be employed in real situations on a large scale.

In future work, we aim to study the automated representation of floor plan images in a graph, to facilitate the use of the application. We plan to integrate the application with smart (e.g., smoke) sensors to discover paths inside the building in as realistic a way as possible. We also intend to carry out studies of complex floor plans, with people simulating the use of a ‘support application’ to find safe emergency exit routes. Additionally, our goal is to apply the developed algorithm and neural network, using deep reinforcement learning (DRL), to explore additional challenges, such as larger floor plans, and, there is an intention to conduct hyperparameter tuning aimed at improving the optimization of the utilized hyperparameters. Furthermore, we plan to investigate other implementations, such as deep reinforcement learning with double Q-learning. 

## Figures and Tables

**Table 1:** Key characteristics of related works.

Reference	Machine Learning	Reinforcement Learning	Real Time	Technique for Representing the Floor Plan (Graph or Matrix)
[8]	Yes	No	Does not specify	No
[23]	Yes	No	No	Yes
[9]	Yes	Yes	Does not specify	No
[7]	Yes	Yes–DRL	Yes	No
**EvacuAI (This study)**	**Yes**	**Yes–DRL–TL**	**Yes**	**Yes**

**Table 2:** Proposed neural network architecture.

Name	Type	In Features	Out Features	Activation
F1	Linear	Observation Size	256	ReLU
F2	Linear	256	128	ReLU
F3	Linear	128	64	ReLU
F4	Linear	64	Qty. of actions	Linear

**Table 3:** Rules for setting the number of episodes.

Condition	Number of Episodes
There is one start node	(Number of edges) × 100
Transfer learning is used	(Number of edges/3) × 500
The number of edges is greater than 40	(Number of edges) × 900
For the other cases	(Number of edges) × 500

**Table 4:** Scenario of the experiments of project 1.

	Project 1	
	With TL	Without TL
Number of Nodes	9	9
Number of Edges	8	8
Observation Space Size	18	18
Number of Episodes: Start Node	1333	800
Number of Episodes: All Nodes	1333	4000

**Table 5:** Scenario of the experiments of project 2.

	Project 1	
	With TL	Without TL
Number of Nodes	31	31
Number of Edges	34	34
Observation Space Size	62	62
Number of Episodes: Start Node	5666	3400
Number of Episodes: All Nodes	5666	17,000

**Table 6:** Scenario of the experiments of project 3.

	Project 1	
	With TL	Without TL
Number of Nodes	34	34
Number of Edges	46	46
Observation Space Size	68	68
Number of Episodes: Start Node	7666	4600
Number of Episodes: All Nodes	7666	41,400

**Table 7:** Summary of project 1 simulations.

Project 1						
	**Simulation 1**		**Simulation 2**	**Simulation 3**		
Blocked Nodes	None		Node 7	Node 4 and Node 5		
Initial Node	None		None	None		
Method	ER with replay init buffer	ER without replay init buffer	Transfer Learning	ER with replay init buffer	ER without replay init buffer	Transfer Learning
Accuracy	100%	100%	87%	100%	100%	100%
Training Time	39 s	33 s	12 s	1 min	51 s	16 s
Inference Time	0.03 s	0.04 s	0.04 s	0.04 s	0.03 s	0.03 s

**Table 8:** Summary of project 2 simulations.

Project 2								
	**Simulation 1**		**Simulation 2**			**Simulation 3**		
Blocked Nodes	None		Node 2			Nodes 11 to 17		
Initial Node	None		None			None		
Method	ER with replay init buffer	ER without replay init buffer	ER with replay init buffer	ER without replay init buffer	Transfer Learning	ER with replay init buffer	ER without replay init buffer	Transfer Learning
Accuracy	3.26%	100%	0%	0%	0%	7%	100%	100%
Training Time	2 min and 29 s	2 min and 53 s	2 min and 53 s	3 min and 32 s	22 s	2 min and 38 s	2 min and 40 s	52 s
Inference Time	0.84 s	0.69 s	0.62 s	0.84 s	0.54 s	0.62 s	0.21 s	0.14 s

**Table 9:** Summary of project 3 simulations.

Project 3							
	**Simulation 1**	**Simulation 2**		**Simulation 3**		**Simulation 4**	
Blocked Nodes	None	Node 22		Node 22		Nodes 4, 5, 6, and 10	
Initial Node	None	None		Node 2		None	
Method	ER without replay init buffer	ER without replay init buffer	Transfer Learning	ER without replay init buffer	Transfer Learning	ER without replay init buffer	Transfer Learning
Accuracy	100%	0%	90%	100%	100%	100%	100%
Training Time	7 min and 14 s	6 min and 43 s	1 min	21 s	20 s	10 min and 21 s	1 min
Inference Time	0.17 s	0.61 s	0.49 s	0.01 s	0.02 s	0.11 s	0.09 s
